# Direct Molecular Fishing of New Protein Partners for Human Thromboxane Synthase

**Published:** 2017

**Authors:** A. V. Svirid, P. V. Ershov, E. O. Yablokov, L. A. Kaluzhskiy, Yu. V. Mezentsev, A. V. Florinskaya, T. A. Sushko, N. V. Strushkevich, A. A. Gilep, S. A. Usanov, A. E. Medvedev, A. S. Ivanov

**Affiliations:** Institute of Bioorganic Chemistry, National Academy of Sciences of Belarus, Akad. Kuprevicha Str. 5 /2, Minsk, 220141, Belarus; Institute of Biomedical Chemistry, Pogodinskaya Street 10, bldg. 8, Moscow, 119121, Russia; Department of Bioengineering, School of Engineering, The University of Tokyo, Tokyo, 108-8639, Japan

**Keywords:** Thromboxane synthase (CYP5A1, TBXAS1), cytochrome P450, surface plasmon resonance, direct molecular fishing, protein partners, isatin

## Abstract

Thromboxane synthase (TBXAS1) catalyzes the isomerization reaction of prostaglandin H2 producing thromboxane A2, the autocrine and paracrine factor in many cell types. A high activity and metastability by these arachidonic acid derivatives suggests the existence of supramolecular structures that are involved in the regulation of the biosynthesis and directed translocation of thromboxane to the receptor. The objective of this study was to identify TBXAS1 protein partners from human liver tissue lysate using a complex approach based on the direct molecular fishing technique, LC-MS/MS protein identification, and protein-protein interaction validation by surface plasmon resonance (SPR). As a result, 12 potential TBXAS1 protein partners were identified, including the components regulating cytoskeleton organization (BBIP1 and ANKMY1), components of the coagulation cascade of human blood (SERPINA1, SERPINA3, APOH, FGA, and FN1), and the enzyme involved in the metabolism of xenobiotics and endogenous bioregulators (CYP2E1). SPR validation on the Biacore 3000 biosensor confirmed the effectiveness of the interaction between CYP2E1 (the enzyme that converts prostaglandin H2 to 12-HHT/thromboxane A2 proantagonist) and TBXAS1 (*K*d = (4.3 ± 0.4) × 10^-7^ M). Importantly, the TBXAS1•CYP2E1 complex formation increases fivefold in the presence of isatin (indole-2,3-dione, a low-molecular nonpeptide endogenous bioregulator, a product of CYP2E1). These results suggest that the interaction between these hemoproteins is important in the regulation of the biosynthesis of eicosanoids.

## INTRODUCTION

Human thromboxane synthase (TBXAS1) belongs to the cytochrome P450 superfamily (CYP5A1). However, it functions differently from “classical” cytochromes P450, which catalyze various monooxygenase reactions, involving redox partners as the electron donors [1]. TBXAS1 catalyzes the reaction of prostaglandin H_2_ (PGH_2_) isomerization, which requires no redox partners and produces thromboxane A_2_ (TXA_2_) [2]. The latter acts as a paracrine and autocrine regulator and is an important mediator of platelet aggregation and contraction of blood vessels, which contributes to increase in blood pressure.

Apart from PGH_2_ isomerization, TBXAS1 catalyzes the alternative PGH_2_ transformation reaction, resulting in its cleavage to 12-hydroxy-5,8,10-heptatrienic acid (12-HHT) and malondialdehyde (MDA) [3]. There is currently no accurate information on the functional role of MDA and 12-HHT. MDA can form adducts with the protein amino groups or polar groups of phospholipids and thus plays a role in the molecular mechanisms of atherosclerosis, cancer, and some genetic diseases [4, 5]. 12-HHT and its metabolites can block the action of leukotriene receptors and act as a partial TXA2 antagonist by enhancing the synthesis of prostacyclin and antagonizing the thromboxane receptor (TXAR) [6, 7]. It is possible that TBXAS1 also performs other functions: catalyze monooxygenase reactions characteristic of cytochrome P450 and involving redox partners.

TBXAS1 was first isolated from human platelets [3] and pig lungs [8]. TBXAS1 is mostly synthesized in prothrombocytes and monocyte precursor hematopoietic stem cells, leukocytes, and macrophages, where TXA_2_ is involved in the regulation of cell differentiation [9]. Synthesis of TBXAS1 was also detected in the cells of lungs, kidneys, the stomach, intestine, spleen, thymus, pancreas, and the liver [10]. TXAR, which belongs to the class of G-protein-coupled receptors (GPCRs), is expressed in many tissues (lung, spleen, liver, uterus, placenta, aorta, heart muscle, intestine, thymus, kidney, brain, and spinal cord) [11]. This may be indicative of other possible functions of TBXAS1 or the versatility of the mechanisms underlying its basic function.

One approach to elucidating the unknown functions of a protein is based on studying its interactions with other proteins whose functions are known [12]. This approach is based on the concept that the functions of the interacting protein partners must be either interrelated or form a single protein complex that performs interrelated functions. The substrate and product of the reaction catalyzed by TBXAS1 are extremely short-living and active lipophilic molecules, whose diffuse transport is complicated, while the TXA2 receptor is located on the outside of the plasma membrane. This suggests the existence of a specific transport mechanism or, most likely, an interaction with the associated protein complexes responsible for the transportation of these short-living compounds.

To date, information on experimental validation of protein-protein interactions (PPI) involving TBXAS1 remains scarse. The BioGRID database includes only two records of identified PPIs involving TBXAS1 (https://thebiogrid.org/112778/summary/homo-sapiens/tbxas1.html?sort=bait): (1) interaction with an eukaryotic elongation factor 1 α-2 (EEF1A2) citing unpublished data [13], and (2) interaction with ubiquitin C (UBC) [14]. It is most likely that both these interactions are nonspecific, since the same BioGRID database includes records of 132 potential interactions of EEF1A2 with 124 partners and 2,332 interaction of UBC with 1,440 partners. In 2016, Meling D.D. cited in the abstract of his dissertation (Protein-protein interactions and mechanistic insights for CYP2J2 and TBXAS1) unpublished data on interaction between TBXAS1 and cytochrome P450 reductase (CPR) (http://hdl.handle. net/2142/ 90774), which undoubtedly may be functionally significant, since CPR is a known protein partner of the microsomal cytochromes P450.

Previously, we developed an integrated approach to the discovery of novel protein partners interacting with a target protein which is based on the use of direct molecular fishing on the affinity sorbent with the immobilized target protein (or peptide) as a ligand, mass spectrometric identification of the isolated proteins, and validation of the potential PPIs by surface plasmon resonance (SPR) [15-17].

The objective of the present study was to search for novel potential TBXAS1 protein partners in the human liver tissue lysate using this approach. As a result, 12 potential TBXAS1 protein partners were isolated on the affinity column with immobilized TBXAS1 using a LC-MS/MS-analysis, one of which was cytochrome P450 (CYP2E1). SPR validation confirmed its interaction with TBXAS1 immobilized on the optical chip and identified another potential protein partner (CYP11B2). SPR experiments with five control cytochromes P450 (CYP2C19, CYP11A1, CYP11B1, CYP3A4, CYP3A5) were negative, indicating the high specificity of the detected PPIs. Since CYP2E1 is involved in the metabolism of various indole derivatives [18], we further investigated the possible influence of the well-known endogenous bioregulator isatin (indole-2,3-dione) [19- 22] on the interaction of CYP2E1 and CYP11B2 with TBXAS1. We found that isatin results in a fivefold increase in affinity of the TBXAS1 • CYP2E1 interaction and does not affect the TBXAS1 • CYP11B2 interaction.

## EXPERIMENTAL

**Protein preparations**

Highly purified ( > 95% according to denaturing polyacrylamide gel electrophoresis (SDS-PAGE)) preparations of the recombinant proteins, TBXAS1, cytochromes P450 (limonene 6-monooxygenase (CYP2C19), steroid-20,22-lyase (CYP11A1), steroid-11β-hydroxylase (CYP11V1), aldosterone synthase (CYP11B2), taurochenodeoxycholate-6α-monooxygenase (CYP3A4), cyclic hydrocarbon hydroxylase (CYP3A5), 4-nitrophenol-2-hydroxylase (CYP2E1), microsomal cytochrome b5 (CYB5A), NADPH-cytochrome-P450-reductase (CPR), NADPH-adrenodoxin reductase (ADR), adrenodoxin (ADX), and ferrochelatase (FECH), SMAD4, RAB27B) were prepared at the Institute of Bioorganic Chemistry (Republic of Belarus) by molecular cloning and heterologous expression in a bacterial system *(E. coli), *followed by purification using metal-affinity and ion exchange chromatography [23, 24]. The preparation of retinol-binding protein 4 (RBP4) was obtained from Cayman chemical (USA).

**Human liver tissue lysate**

Human liver tissue samples were obtained from the ILSbio LLC (www.ilsbio.com). The lysate was prepared by homogenization of a 100-mg tissue liver sample in a Potter mortar with 1 mL of the CellLytic Mammalian Tissue Lysis/Extraction Reagent (Sigma, USA) and 10 μL of a protease inhibitor cocktail (Sigma, USA). After centrifugation at 13,400 *g *and 4°C for 25 min, the supernatant was collected, glycerol was added to a final concentration of 25%, and the resulting solution was stored at –80°C. The total protein concentration in the lysate samples was 10–20 mg/mL, as determined spectrophotometrically using a Bradford assay.

**Direct molecular fishing**

An affinity sorbent with covalently immobilized TBXAS1 as the bait protein was prepared by covalent protein binding to CNBr-Sepharose 4B (GE Healthcare, USA) according to the manufacturer’s protocol. It was found that the 0.5 mg/1 g protein to sorbent ratio was optimal for binding of the used TBXAS1 preparation to the sorbent. The remaining active groups of the sorbent were inactivated by incubation in a buffer containing 100 mM Tris-HCl (pH 7.4) and 150 mM NaCl. Direct molecular fishing was carried out in the original microcolumn (volume 200 µL) filled with the affinity sorbent. In the control experiments, a similar microcolumn filled with “empty” (no bait protein) inactivated CNBr-Sepharose 4B was used. HBS-EP+ buffer (10 mM HEPES (pH 7.4), 150 mM NaCl, 3 mM EDTA, 0.05% Tween 20) passed through the microcolumn at a flow rate of 50 µL/min at 15°C was used as a running buffer. Affinity isolation of the TBXAS1 protein partners was carried out by passing 2 mL of lysate (0.5 mg/mL of protein) twofold diluted with the running buffer through the column for 80 minutes using the 10 AKTA Purifier (GE Healthcare, USA) system. Proteins from the lysate bound to the sorbent were eluted with 4% HCOOH (pH 2.5) at a flow rate of 50 μL/min for 100 min. The total protein content in the eluates determined by the Bradford assay was 25–35 μg/mL (average value 30 μg/mL). The experiments on the affinity isolation of potential TBXAS1 protein partners were repeated in triplicates.

**LC-MS/MS-analysis**

Special sample preparation was used for the mass-spectrometric identification of the proteins. An aliquot containing 30 μg of total protein was sampled from each chromatographic fraction and subjected to the standard trypsinolysis procedure with preliminary reduction and alkylation of the sulfhydryl groups of the proteins. All procedures were carried out in Vivaspin 500 Centrifugal Concentrators, 10 kDa MWCO (GE Healthcare, USA) using the FASP method [25]. A lyophilized trypsin preparation obtained from porcine pancreas (activity 15600 IU/mg, V5111, Promega, USA) was used for trypsin digestion of the proteins. Mass-spectrometric analysis of the samples was carried out in three technical replicates using a Agilent 1200 chromatograph and Agilent 6300 mass-detector with Ion Trap LC/MS (Agilent Technologies, USA). Peptides were separated using a reversed-phase HPLC-column ZORBAX Extend-C18 (2.1 × 150 mm, 1.8 μm) (Agilent Technologies, USA) in a gradient of solvent A (0.2% formic acid in water) and Solvent B (0.2% formic acid in acetonitrile) for 55 min at a flow rate of 350 μL/min. The sample volume applied to the column was 15 μL (~ 7–8 mg of material). The gradient was as follows: from 0 to 20% of solvent B in 5 minutes, from 20 to 80% in 40 minutes, from 80 to 95% in 5 min, and 95% for 5 min. Column temperature was 50°C. Mass spectra were acquired in the positive ionization mode (APESI-ionization) with the following parameters: gas temperature 400°C, gas flow rate 9 L/min, capillary voltage 2 kV, and fragmentor voltage 360 V. The mass analyzer was operated in the auto-MS/MS-mode with the following parameters: m/z range from 50 to 2200 m/z, fragmentation energy was calculated according to the following formulas: (3.1(m/z)/100 + 1.0) V for z = 2 and (3.6(m/z)/100 – 4.8) V for z ≥ 3. The proteins were identified using the Mascot software (www.matrixscience.com) and SwissProt database (www.uniprot. org). The following search parameters were used: proteolytic enzyme trypsin, acceptable mass deviation of monoisotopic peptides ± 2.6 Da, acceptable MS/MS deviation ± 0.6 Da, acceptable number of omitted trypsin cleavage sites is 2, variable modification – “oxidized methionine,” and fixed modifications – “carbamidomethyl.” The resulting list of reliably detected proteins included only those proteins which were identified in three technical replicates with a significance of 0.01 and Mascot Score > 50.

**Surface plasmon resonance (SPR)**

PPIs were analyzed on the four-channel optical biosensor Biacore 3000 (GE Healthcare, USA), whose operation is based on the surface plasmon resonance effect controlled by the Biacore Software v. 1.0. Biosensor signals were recorded in resonance units, RU (1 RU corresponds to binding of about 1 pg of the protein on the optical chip surface). The values of the equilibrium dissociation constants *(K*_d_), association rate constants *(k*_on_), and dissociation rate constants *(k*_off_) of the complexes were calculated using the BiaEvaluation v. 4.1 software package.

TBXAS1 was immobilized by the formation of covalent bonds between the carboxyl groups on the surface of the optical chip CM5 and the free amino groups of the protein. For this purpose, we used the Amine Coupling Kit (GE Healthcare, USA). The TBXAS1 sample (50 μg/mL) in 10 mM acetate buffer (pH 5.0) was injected at a flow rate of 5 μL/min for 20 min. The TBXAS1 immobilization level in the working channel of the optical biosensor averaged 7,500 RU (7.5 ng/mm2).

**Fig. 1:**
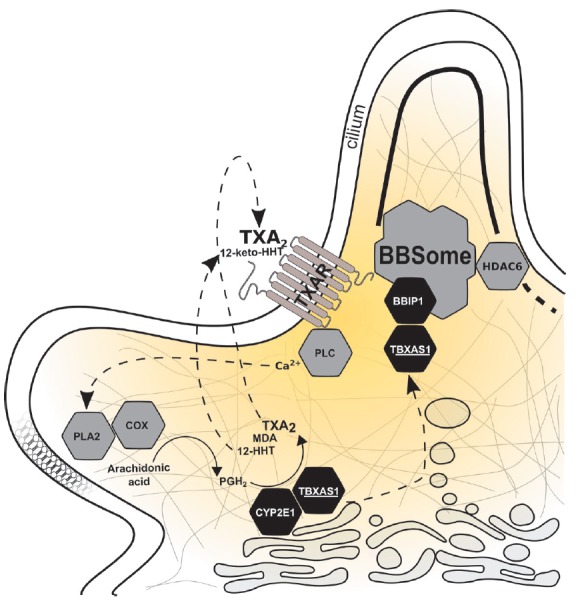
Schematic representation of TXA2 biosynthesis supplemented by our experimental results. PLC – phospholipase C, PLA2 – phospholipase A_2_, COX – cyclooxygenase. Biosynthesis of TXA_2_ begins with the release of arachidonic acid from membrane phospholipids assisted by PLA2. Then, COX catalyzes the transformation of arachidonic acid into prostaglandin H2, which is then metabolized by TBXAS1 to form TXA_2_, 12-HHT, and MDA. At the same time, prostaglandin H2 is transformed by CYP2E1 to 12-HHT and MDA. TXA_2_ binding to TXAR causes signal transmission via the inositol phosphate pathway with PLC activation and mobilization of intracellular Ca^2+^, which has a stimulating effect on PLA2. Further, 12-HHT is metabolized by 15-hydroxyprostaglandin dehydrogenase to form 12-keto-HHT, which has a partial antagonistic effect on TXAR. TBXAS1 also presumably interacts with BBIP1, which is a component of the protein transport complex of cilia (BBSome). BBIP1 can influence the stability of the microtubulin cytoskeleton, indirectly inhibiting HDAC6 (microtubule deacetylase).

Interactions of test proteins with the immobilized TBXAS1 were recorded in real time mode while injecting protein samples at a concentration ranging from 50 nM to 5 μM through the control channel (without the protein) and then through the channel with immobilized TBXAS1 for 10 min at a flow rate of 5 μL/min. Each measurement was followed by a regeneration of the optical chip surface by injecting buffer containing 2 M NaCl and 0.4% CHAPS for 30 s at a flow rate of 20 μL/min. All measurements were performed at least 4 times, which provided adequate accuracy and reproducibility (CV value was less than 10%). In the experiments assessing the possible impact of nonpeptide low-molecular-weight endogenous bioregulator isatin (2,3-dioxoindole) on PPIs involving TBXAS1, isatin was added to the samples of analyzed proteins at a final concentration of 100 μM and the mixture was incubated for 15 min.

## RESULTS AND DISCUSSION

An integrated approach based on direct molecular fishing on an affinity sorbent with a 4B target protein immobilized on CNBr-Sepharose, mass-spectrometric identification of isolated proteins, and validation of PPIs with SPR enables the isolation and identification of 12 potential TBXAS1 protein partners from the lysates of human liver tissue (*Table*). To date, the scientific literature still provides no information on the interaction between these proteins and TBXAS1. However, some assumptions about their possible functional relationship with TBXAS1 can be made. For example, the BBIP1 protein (BBSome component of the transport protein complex of cilia) is involved in the regulation of cellular cytoskeleton stability [26, 27]. Information on ANKMY1 is available only at the transcript level. However, its structure includes ankyrin repeats, which form one of the most common interfaces for PPIs. These repeats were found in proteins characterized by various functions [28]. Based on this fact, we assumed that ankyrin repeats of ANKMY1 can specifically recognize certain structural motifs of TBXAS1, facilitating interaction between these proteins.

**Fig. 2:**
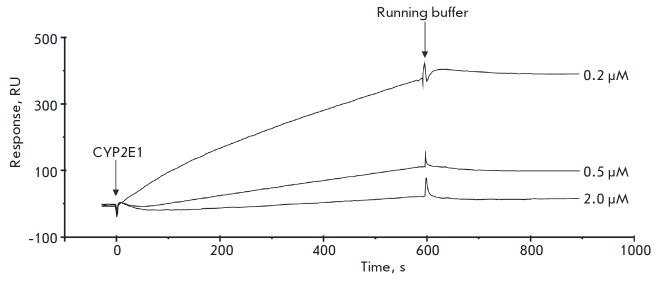
Typical sensorgrams of the interaction between various concentrations of CYP2E1 and TBXAS1 immobilized on a CM5 optical chip

However, it should be noted that proteins identified by direct molecular fishing can only be considered as potential protein partners of TBXAS1, since not only real partner proteins, but also simultaneously “fished” extraneous proteins composing micelles or supramolecular complexes may be isolated from the lysate due to the features of this techniques [17]. Among our “fished” proteins (*table*), these are SERPINA1, SERPINA3, APOH, FGA, and FN1, which are involved in the blood clotting cascade [29, 30], as well as serum proteins (HP, SAA1, CP), which can have a high nonspecific adsorption level.

**Fig. 3:**
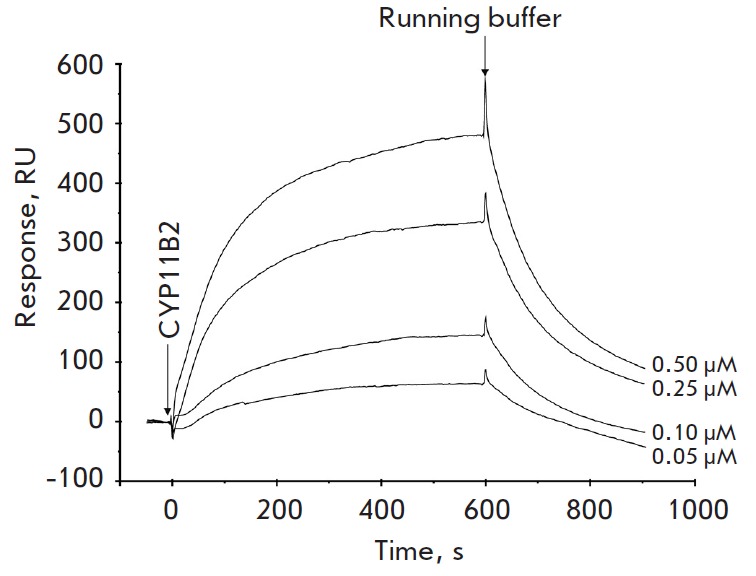
Typical sensorgrams of the interaction between various concentrations of CYP11B2 and TBXAS1 immobilized on a CM5 optical chip

The presence of CYP2E1, which belongs to the cytochromes P450 superfamily, in the list of “fished” proteins is of particular interest. A functional relationship between CYP2E1 and TBXAS1 may be important in the context of the complementary enzymatic conversion reactions of common substrates. As it is known, CYP2E1 is characterized by broad substrate specificity and a broad tissue localization profile, including the liver [31]. For example, CYP2E1 can oxidize arachidonic acid (via ω-1-hydroxylation) and prostaglandin H2 [32] to form side metabolites, which, in turn, are formed in the prostaglandin H2 to thromboxane A2 isomerization reactions. 12-keto-HHT is a further metabolite of one of the reaction products and can influence TXA2 by increasing prostacyclin production and antagonistic action on TXAR [4, 5]. Therefore, colocalization of TBXAS1, synthesizing thromboxane A2, and CYP2E1 could serve as an additional mechanism regulating the effectiveness of enzymatic conversion of common substrates. On the other hand, oligomerization of various cytochromes P450 can also lead to change in the catalytic parameters of enzymatic reactions: e.g., the affinity of the enzymes to the substrate [33]. Schematic representation of a TXA2 biosynthesis system complemented by our experimental data is shown in *Fig. 1*.

**Fig. 4:**
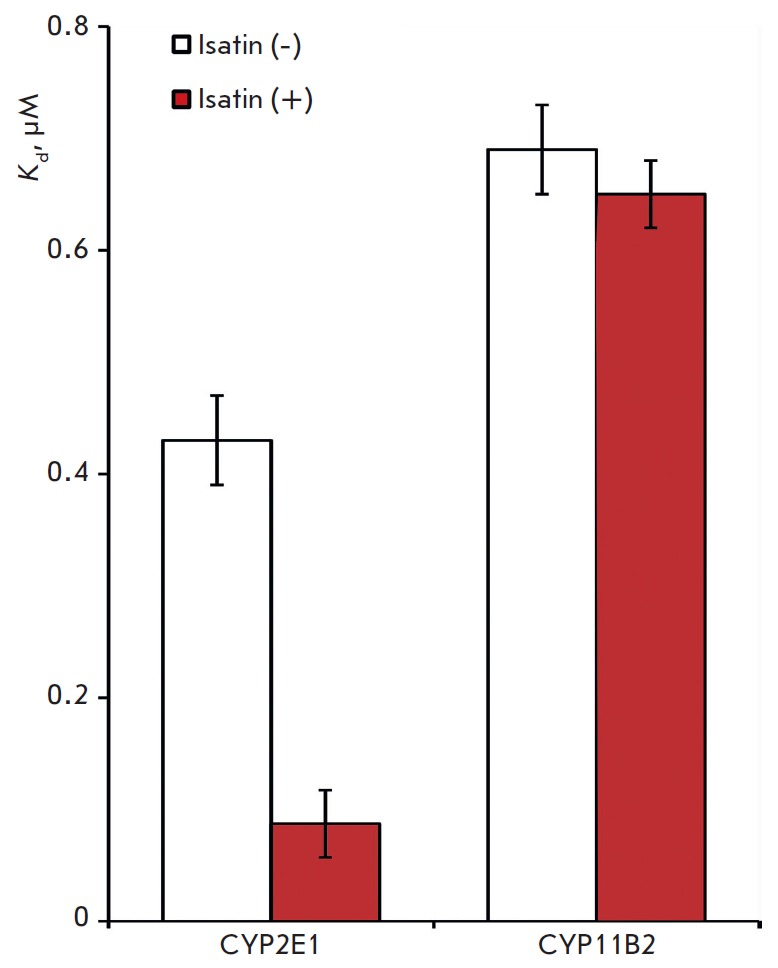
Diagram representation of equilibrium dissociation constant (*K*_d_) values of the TBXAS1•CYP2E1 and TBXAS•CYP11B2 complexes in the absence and presence of 100 μM of isatin; M ± m, n = 3

We confirmed the formation of the heteromeric TBXAS1 • CYP2E1 complex in direct SPR experiments (*Fig. 2*). The specificity of the TBXAS1 and CYP2E1 interaction was tested by running control SPR experiments using both microsomal (CYP2C19, CYP3A4, CYP3A5) and mitochondrial (CYP11A1, CYP11B1, CYP11B2) cytochromes P450 as analytes. Other well known cytochrome P450 protein partners (CYB5A, CPR, ADR, ADX) and several proteins unrelated to the cytochrome P450 monooxygenase system (FECH, SMAD4, RAB27B, RBP4) were also used for the specificity test. It was shown that all protein analytes, except for CYP11B2 (*Fig. 3*), did not bind to TBXAS1 immobilized on the optical chip even at micromolar concentrations. A similar control experiment using TBXAS1 as a protein analyte showed no dimerization or oligomerization process. Thus, we can confidently state that the interaction of CYP2E1 and CYP11B2 with TBXAS1 is highly specific.

**Table:** Mass-spectrometric identification of the proteins in eluates from chromatographic microcolumns filled with an affinity sorbent

S/N	Gene	Protein	MW, Da	Uniprot number^a^	Score^b^	Peptides^c^	emPAI^d^
Sorbent with immobilized thromboxane (only Test). (TBXAS1 potential protein partners)							
1	FGA	Fibrinogen alpha chain	95656	P02671	97	19 (2)	0.04
2	FN1	Fibronectin	266052	P02751	95	14 (2)	0.01
3	CP	Ceruloplasmin	122983	Q24478	85	10 (5)	0.03
4	SERPINA3	Alpha1-antichymotrypsin	47792	P01011	67	9 (3)	0.08
5	SAA1	Serum amyloid A1 protein	13581	P0DJI8	67	7 (3)	0.29
6	CYP2E1	Cytochrome P450 2E1	56849	P05181	61	11 (4)	0.12
7	ANKMY1	Ankyrin repeat and MYND domain-containing protein 1	107101	Q9P2S6	59	25 (7)	0.03
8	ACTB	Actin, cytoplasmic 1	42052	P60709	56	17 (2)	0.13
9	BBIP1	BBSome-interacting protein 1	10557	A8MTZ0	54	10 (4)	0.38
10	SERPINA1	Alpha 1-antitrypsin	46878	P01009	50	3 (3)	0.13
11	APOH	Beta2-glycoprotein 1	39584	P02749	52	3 (3)	0.09
12	HP	Haptoglobin	45861	Q61687	50	42 (6)	0.21
Sorbent without protein immobilization (only Control)							
1	ACY1	Aminoacylase-1	46084	Q03154	59	17 (3)	0.08
2	ADH1A	Alcohol dehydrogenase 1A	40745	P07327	189	85 (19)	0.42
3	SLC25A4	ADP/ATP translocase 1	33271	P12235	59	16 (5)	0.24
4	SLC25A5	ADP/ATP translocase 2	33059	P05141	71	27 (5)	0.11
5	MAOB	Amine oxidase [flavin-containing] B	59238	P27338	147	31 (8)	0.20
6	ASL	Argininosuccinate lyase	51910	P04424	89	9 (3)	0.07
7	ASS1	Argininosuccinate synthase	46786	P00966	59	48 (6)	0.17
8	ATP5B	ATP synthase subunit beta, mitochondrial	56525	P25705	108	27 (4)	0.07
9	ATP5C1	ATP synthase subunit gamma, mitochondrial	33032	P36542	95	16 (4)	0.24
10	CALR	Calreticulin	48283	P27797	68	10 (4)	0.25
11	HSPD1	60 kDa heat shock protein, mitochondrial	61187	P10809	126	28 (7)	0.13
12	CPS1	Carbamoyl-phosphate synthase [ammonia],mitochondrial	165975	P31327	249	119 (27)	0.17
13	DEFA1	Neutrophil defensin 1	10536	P59665	58	8 (5)	0.38
14	FBP1	Fructose-1,6-bisphosphatase 1	37218	P09467	78	4 (3)	0.10
15	FABP1	Fatty acid-binding protein, liver	14256	P07148	184	29 (17)	1.08
16	GAPDH	Glyceraldehyde-3-phosphate dehydrogenase	36201	P04406	169	23 (5)	0.22
17	AGL	Glycogen debranching enzyme	176819	P35573	77	18 (7)	0.06
18	SHMT1	Serine hydroxymethyltransferase, cytosolic	53619	P34896	88	16 (5)	0.14
19	HSPA5	78 kDa glucose-regulated protein	72402	P11021	75	19 (4)	0.11
20	GSTA1	Glutathione S-transferase A1	25672	P08263	94	74 (13)	1.29
21	IDH1	Isocitrate dehydrogenase [NADP] cytoplasmic	46915	O75874	274	15 (9)	0.17
22	IDH2	Isocitrate dehydrogenase [NADP], mitochondrial	51333	Q8IQA7	105	10 (4)	0.15
23	LDHA	L-lactate dehydrogenase A chain	36950	P00338	240	9 (8)	0.21
24	NONO	Non-POU domain-containing octamer-binding protein	54311	Q15233	102	27 (6)	0.14
25	PGM1	Phosphoglucomutase-1	61696	P36871	120	10 (6)	0.12
26	SFPQ	Splicing factor, proline- and glutamine-rich	76216	P23246	106	27 (7)	0.10
27	ACAT1	Acetyl-CoA acetyltransferase, mitochondrial	45456	P24752	60	11 (4)	0.17
28	TPI1	Triosephosphate isomerase	31057	P60174	150	12 (4)	0.26
29	UGT2B10	UDP-glucuronosyltransferase 2B10	61190	P36537	55	8 (3)	0.06
30	UGP2	UTP-glucose-1-phosphate uridylyltransferase	57076	Q16851	72	15 (4)	0.13
Control - upper line, Test - bottom line							
1	UGP2	Alcohol dehydrogenase 1B	40684	P00325	408	117 (34)	1.02
					90	53 (6)	0.30
2	ADH4	Alcohol dehydrogenase 4	41108	P08319	222	36 (16)	0.42
					85	13 (8)	0.30
3	ALB	Serum albumin	71317	P02768	2790	360 (150)	1.90
					959	186 (50)	0.66
4	ALDH2	Aldehyde dehydrogenase, mitochondrial	56859	P05091	400	33 (15)	0.29
					94	7 (3)	0.07
5	ALDOB	Fructose-bisphosphate aldolase B	39961	P05062	167	25 (8)	0.31
					220	28 (8)	0.09
6	APOA1	Apolipoprotein A-I	30759	P02647	98	12 (6)	0.42
					53	25 (6)	0.59
7	ATP5F1	ATP synthase subunit b, mitochondrial	28947	P24539	192	17 (10)	0.45
					97	13 (5)	0.28
8	ATP5L	ATP synthase subunit g, mitochondrial	11421	O75964	265	7 (6)	0.35
					157	5 (5)	0.35
9	DCXR	L-xylulose reductase	26182	Q7Z4W1	194	27 (6)	0.15
					161	8 (5)	0.15
10	DECR1	2,4-dienoyl-CoA reductase, mitochondrial	36330	Q16698	250	24 (11)	0.22
					222	13 (8)	0.10
11	HSD17B4	Peroxisomal multifunctional enzyme type 2	80092	P51659	1754	179 (87)	1.26
					112	16 (5)	0.09
12	SORD	Sorbitol dehydrogenase	38927	Q00796	171	17 (13)	0.44
					73	7 (4)	0.10
13	CES1	Liver carboxylesterase 1	62766	P23141	88	28 (4)	0.06
					83	20 (4)	0.06
14	HBA1	Hemoglobin subunit alpha	15305	P69905	70	48 (10)	2.90
					82	16 (6)	0.25
15	HBB	Hemoglobin subunit beta	16102	P68871	153	30 (11)	0.54
					222	29 (14)	0.54
16	HMGCS2	Hydroxymethylglutaryl-CoA synthase, mitochondrial	57113	P22791	249	26 (13)	0.37
					98	10 (3)	0.07
17	HRG	Histidine-rich glycoprotein	60510	P04196	60	14 (6)	0.13
					52	9 (5)	0.13
18	PHB2	Prohibitin-2	33276	Q99623	107	11 (4)	0.11
					99	7 (3)	0.11
19	ACAA2	3-ketoacyl-CoA thiolase, mitochondrial	42354	P42765	82	20 (6)	0.18
					71	15 (3)	0.18
20	TF	Serotransferrin	79294	P02787	138	20 (6)	0.15
					142	22 (7)	0.20
21	SLC25A1	Tricarboxylate transport protein, mitochondrial	34333	P53007	70	9 (4)	0.23
					54	10 (3)	0.23

^a^ - Numbers in the Uniprot database (http://www.uniprot.org).

^b^ - The reliability of peptide identification by mass spectrometry (MASCOT score).

^c^ - The number of MASCOT peptides; the number of unique peptides (in parentheses).

^d^ - emPAI, Exponentially Modified Protein Abundance Index.

The names of the identified proteins are listed in the same form as they appear in the Uniprot database used for their identification.

The calculated *K*d values of TBXAS1 • CYP11B2 and TBXAS1 • CYP2E1 complex formation were (6.9 ± 0.3) × 10^-7^ M and (4.3 ± 0.4) × 10^-7^ M, respectively. These values are comparable to *K*_d_ of the complexes of various cytochromes P450 with their functional partners (CPR, CYB5A, ADX) [23, 34-37]. It is important to note that, while the difference in the *K*_d_ values of complex formation is about twofold, TBXAS1 • CYP11B2 and TBXAS1 • CYP2E1 interactions are very different in their kinetic parameters. Association and dissociation of TBXAS1 • CYP2E1 occur about an order of magnitude slower compared to TBXAS1 • CYP11B2. Association rate constants *(k*_on_) are at a 10-fold difference, and dissociation rate constants of the complexes *(k*_off_) are at a 15-fold difference.

The revealing of specific TBXAS1 • CYP11B2 complex formation was a new and unexpected result, since CYP11B2 was not identified as a protein partner of TBXAS1 used as a bait in the experiments on molecular fishing from liver tissue lysate (*Table*). These data are quite comparable, they are not due to the false-negative results of molecular fishing, and can be explained in terms of the tissue-specific CYP11B2 expression profile (preferential expression in the adrenal tissue), which appears from the information in the open Internet resources Proteinatlas (http://www.proteinatlas.org) and Genecards (http://www.genecards.org) and publications [38]. It is currently difficult to deduce the functional consequences and causes of this PPI, so this paper reports only on the fact of experimental confirmation of a direct interaction between TBXAS1 and CYP11B2.

It is known that indole is oxidized to isatin by some cytochromes P450 (CYP2A6, CYP2C19, and CYP2E1) which are responsible for the metabolism of various xenobiotics [18]. Isatin is an endogenous bioregulator with a wide range of biological and pharmacological activities which are implemented when it interacts with many intracellular isatin-binding proteins [19-22, 39-41]. Since CYP2E1 turned out to be one of the proteins that interact with TBXAS1, we assumed that isatin can affect TBXAS1 • CYP2E1 complex formation. This hypothesis was tested via SPR analysis of the interaction between TBXAS1 and CYP2E1 in the absence and presence of isatin. We found that isatin really affects TBXAS1 • CYP2E1 complex formation, but it has no effect on the TBXAS1 • CYP11B2 interaction (*Fig. 4*) used as a control*. *The effect of the fivefold increase in the affinity of TBXAS1 • CYP2E1 in the presence of isatin is due to both a twofold increase in *k*_on_ values and a 2.5-fold decrease in *k*_off_.

## CONCLUSIONS

TBXAS1 potential protein partners were isolated from a human liver tissue lysate by direct molecular fishing and mass-spectrometric identification. Using the SPR biosensor technique, it was for the first time shown that TBXAS1 interacts with cytochrome P450 CYP2E1 and CYP11B2, while the affinity of TBXAS1•CYP2E1 complex formation is fivefold higher in the presence of low-molecular-weight non-peptide endogenous bioregulator isatin (2,3-dioxindole). Overall, our results suggest that TBXAS1 has other functions, such as participation in the functioning of the cytoskeleton and regulation of the biosynthesis of biologically active molecules.

## Abbreviations

PPI: Protein–protein interaction
SPR: surface plasmon resonance
k_on_: association rate constant
k_off_: dissociation rate constant
K_d_: equilibrium dissociation constant
